# Electrospun Nanofibers/Nanofibrous Scaffolds Loaded with Silver Nanoparticles as Effective Antibacterial Wound Dressing Materials

**DOI:** 10.3390/pharmaceutics13070964

**Published:** 2021-06-26

**Authors:** Sibusiso Alven, Buhle Buyana, Zizo Feketshane, Blessing Atim Aderibigbe

**Affiliations:** Department of Chemistry, University of Fort Hare, Alice 5700, Eastern Cape, South Africa; 201214199@ufh.ac.za (S.A.); 201301441@ufh.ac.za (B.B.); 201504226@ufh.ac.za (Z.F.)

**Keywords:** wound treatment, wound dressings, scaffolds, nanofibers, nanofibrous membranes, nanofibrous mats, and nanoparticles

## Abstract

The treatment of wounds is expensive and challenging. Most of the available wound dressings are not effective and suffer from limitations such as poor antimicrobial activity, toxicity, inability to provide suitable moisture to the wound and poor mechanical performance. The use of inappropriate wound dressings can result in a delayed wound healing process. Nanosize range scaffolds have triggered great attention because of their attractive properties, which include their capability to deliver bioactive agents, high surface area, improved mechanical properties, mimic the extracellular matrix (ECM), and high porosity. Nanofibrous materials can be further encapsulated/loaded with metal-based nanoparticles to enhance their therapeutic outcomes in wound healing applications. The widely studied metal-based nanoparticles, silver nanoparticles exhibit good properties such as outstanding antibacterial activity, display antioxidant, and anti-inflammatory properties, support cell growth, making it an essential bioactive agent in wound dressings. This review article reports the biological (in vivo and in vitro) and mechanical outcomes of nanofibrous scaffolds loaded with silver nanoparticles on wound healing.

## 1. Introduction

Wounds are generally defined as injury in the epithelial layer of the skin [1,2]. Wounds are categorized as chronic and acute wounds according to the nature and duration of their healing process [3]. Examples of chronic wounds include burn wounds, diabetic wounds, diabetic foot ulcers, leg ulcers, and decubitus ulcers [4]. Acute wounds, on the other hand, usually heal at an expected and predictable time, depending on the extent, size, and depth of injury in the dermis and epidermis lining of the skin. Acute wounds can result in chronic wounds due to neglected wound treatment, diseases (such as diabetes, cancer, etc.), malnutrition, obesity, smoking, and microbial infection [5,6,7,8,9,10]. The cost of wound management is expensive, and the healthcare system spends more than $25 billion every year globally [11,12]. Wounds can also be categorized based on their depth as superficial wounds (damage of the epidermal lining), partial-thickness wounds (affecting both the epidermal and dermal layer), and full-thickness wounds (damage to both layers and other deep tissues and subcutaneous fat). The phases of wound healing must be considered to determine the appropriate management or treatment of injuries [13].

The wound healing process involves four sequential phases: haemostasis, inflammatory, proliferative, and maturation phase [14,15]. The haemostasis phase occurs immediately after the injury and is characterized by coagulation of exudate and blood clotting induced by platelets and fibrinogen to control bleeding [16]. The inflammatory phase usually occurs simultaneously with the haemostasis phase. In this phase, the wound is protected from bacterial infections by proteases, neutrophils, and reactive oxygen species (ROS) [17]. The proliferative phase is where the wound is covered completely by epithelial cells by granulating tissue [18,19]. The last stage is the maturation phase, also known as remodeling, the fibroblasts cover the wound site as the new epidermal skin layer [18,20]. Various wound dressings materials can be employed for each phase of the wound healing process, depending on their unique properties [20].

Most of the currently utilized wound dressings are prepared from polymers, either natural or synthetic. Some examples of natural polymers used to prepare wound dressings include chitosan, alginate, dextran, cellulose, chitin, elastin, etc. [21]. The dressing materials prepared from these polymers usually suffer from poor mechanical properties. This limitation can be easily overcome by crosslinking with synthetic polymers such as poly (α-esters), poly (lactic acid) (PLA), poly (glycolic acid) (PGA), poly (D,L-glycolic-co-lactic acid) (PLGA), poly (ԑ-caprolactone) (PCL)], poly(vinyl pyrrolidone) (PVP), poly(ethylene oxide) (PEO)/poly (ethylene glycol) (PEG), poly(vinyl alcohol) (PVA), poly(hydroxyethyl methacrylate) (PHEMA), polyurethanes (PUs) [22]. There are different forms of wound dressing materials that can be prepared from these polymers, such as films, sponges, hydrogels, bandages, exosomes, foams, wafers, nanofibers, and nanofibrous materials (including nanofibrous mats, patches, films, membranes, etc.) [20].

Most of the above-mentioned wound dressings suffer from poor antimicrobial properties [23]. Nanoparticles display excellent antibacterial activity that can be used in wound dressings [24]. These nanomaterials can be encapsulated in wound dressings to improve the wound healing process. Examples of nanoparticles loaded in wound dressings include metal-based nanoparticles, polymeric nanoparticles, and lipid-based nanoparticles [24]. This review article is focused on the biological outcomes of nanofibrous materials (i.e., nanofibrous mats and nanofibrous membranes) encapsulated with metal-based silver (Ag) nanoparticles as potential antibacterial wound dressings.

## 2. Classification of Wound Dressings

Properties of ideal wound dressing materials that must be considered during their preparation are high porosity, high swelling capacity, good water vapour transmission rate, good exudate-absorbing ability, good biological properties (e.g., antibacterial or anti-inflammatory), excellent mechanical properties (such as good tensile strength, elasticity, flexibility, and spreadability), drug loading capacity, and ability to provide a moist environment for the acceleration of wound healing process [25,26,27,28,29]. Most of the currently used wound dressings do not possess some of these properties. Wound dressings that are currently used are classified as traditional wound dressings, skin substitutes, interactive dressings, and bioactive dressings (Table 1) [30]. Wound dressings effective for restoring injured skin cells are known as skin substitutes. Autografts, acellular xenografts, and autografts are examples of skin substitutes. Their shortcomings are the transmission of infections, short survival period in the wound environment, and a high possibility of host rejection [29].

Traditional dressings are called passive wound dressings. Traditional dressings protect the wound from impurities and other foreign substances, control bleeding, absorb wound exudates, cover and cushion the wound, and offer a dry environment for the wounds [30]. Examples of traditional dressings include gauze, bandages, wool dressings, and plaster. The limitations of passive dressings are the need for frequent changes during the wound healing process due to the absorbed wound exudates resulting in skin damage, pain, and bacterial invasion [3]. Interactive dressings are useful in protecting the injury from microbial infections that delay the wound healing process. They also provide moisture to enhance wound healing mechanism, improve re-epithelialization and granulation, and enhance water vapour transmission rate with good mechanical properties [31]. They are prepared from either natural or synthetic polymers such as PVA, PLGA, PEO, PU, gelatin, cellulose, alginate, chitin, and chitosan [32]. Examples of interactive dressings include films, membranes, sprays, sponges.

Bioactive wound dressings are wound dressings that are utilized for the delivery of bioactive agents. Bioactive agents that can be delivered by these dressings include growth factors, nutrients, plant extract, and stem cells. These wound dressings are prepared from natural polymers such as pectin, collagen, alginate, hyaluronic acid, silk fibroin, chitosan, elastin, cellulose, and collagen. Recently, there have been reports on bioactive dressings that are patient compliance, biodegradable, and biocompatible. Hydrogels, wafers, foams, sponges, transdermal patches, nanofibers, etc., are examples of bioactive wound dressings [33].

**Table 1:** Classifications of wound dressings with their functions and limitations.

Types of Wound Dressings	Functions in Wound Healing Applications	Limitations	References
Skin substitutes	Replace damaged skin, less vascularized wound bed required, reduce scar formation, and increases the dermal component of healed wound	Host rejection, possibilities of infection transmission, and limited life-span at the wound site	[30]
Traditional/passive dressings	Protect the wound from foreign contamination, controls bleeding, covers the wound, absorb exudates, and provide cushion	Requires frequent changing, causes re-skin damage, and pain	[31]
Interactive dressings	Offer moist environment at the wound site, promotes re-epithelialization, act as a barrier against infection, good mechanical properties, and enhance water vapor transmission	Limited antibacterial activity	[32]
Bioactive dressings	Biocompatible, patient compliant, biodegradable, useful as delivery systems for bioactive agents.	No obvious limitations	[34]

## 3. Electrospinning Technique and Properties of Nanofibrous Materials as Wound Dressings

There are various types of techniques employed to prepare nanofibers and nanofibrous scaffolds. Those techniques include melt-blowing, phase separation, template synthesis, self-assembly, etc. However, the most useful and commonly employed technique is electrospinning [34]. The electrospinning method has been utilized since the 1890s [35]. The technique uses electrostatic force to pull the fibres from the droplet produced at the tip of a spinneret. Different experiments have been performed to evaluate suitable parameters for electrospinning nanofibers with good physicochemical properties for biomedical applications [36]. The development of nanofibrous scaffolds during electrospinning is influenced by the following three parameters: solution parameters (e.g., solution viscosity, conductivity, and surface tension), process parameters (e.g., flow rate, applied voltage, the distance between collector and tip, and electric field stimulated by the collector), ambient parameters (e.g., humidity and temperature) [34]. The electrospinning technique can produce continuous fibers utilizing a broad range of materials such as polymers and their composites. This method can produce nanofibers/nanofibrous scaffolds with the average diameter ranging from micron to nanosized diameter [37]. Some examples of nanofibrous scaffolds include nanofibrous mats, nanofibrous membranes, nanofibrous webs, nanofibrous patches, etc. [38].

There are several biomedical applications of electrospun nanofibers and nanofibrous scaffolds, such as wound dressings, drug delivery systems, tissue regeneration, etc. [39]. The nanofibrous scaffolds that are based on polymers play a vital role in wound healing applications. It is very important to select appropriate polymers for the preparation of nanofibrous scaffolds that would be appropriate with the required features for wound dressing. Natural polymers such as chitosan, silk, cellulose, and hyaluronic acid (HA), and collagen have been electrospun for applications in drug delivery [40]. Recent studies of the biological and physical properties of natural polymers have demonstrated that biopolymers possess good biocompatibility and are very useful in the development of nanofibrous scaffolds for wound healing [41].

Electrospun nanofibrous materials made from synthetic polymers have demonstrated excellent mechanical properties compared to biopolymers. Furthermore, synthetic polymers are very soluble in a wide range of solvents, which promotes their use in the electrospinning method [42]. Biopolymers are combined with synthetic polymers to control the degradation rate, improve the morphology and mechanical properties of the nanofibrous scaffolds. The electrospun nanofibrous materials have been used to accelerate the wound healing process and to prevent post-surgical infections with controlled and sustained drug release profiles [41]. The poly (ethylene-co-vinyl alcohol) (EVOH) nanofibrous material encapsulated with silver (Ag) nanoparticles has been utilized as a wound dressing to inhibit inflammation by controlled release of Ag nanoparticles [43].

The parameters used in electrospinning influence the generation of bead-free and smooth electrospun fibers. To better understand the fabrication and electrospinning technique of polymeric nanofibers, it is crucial to understand the effects of the regulated parameters [44].

### 3.1. Effect of Flow Rate

The flow of polymeric solutions determines the morphology of the electrospun nanofibers through the metallic needle’s tip. Critical flow rate is used to prepare uniform beadless electrospun nanofibers for a polymeric solution. When the flow rate is increased above the critical value, it leads to bead formation. Increasing the flow rate beyond a critical value also results in increased pore size and fiber diameter due to incomplete drying of the nanofiber jet during the flight between the needle tip and metallic collector [45]. The flow rate affects the nanofiber diameter and formation, and a minimum flow rate is preferred to maintain a balance between the replacement and leaving the polymeric solution of that solution with a new one during the formation of the jet [45,46]. The formation of ribbon-like structures and beads with an increased flow rate is attributed to the non-evaporation of low stretching and solvent of the solution in the flight between the metallic collector and needle. The diameter of the nanofibers increased with an increase in the flow rate [47]. The presence of the unspun droplets is attributed to the influence of the gravitational force. Using the upward-facing nozzle setup reduces the tendency of unspun droplets and beaded fibers formation, as opposed to downward-facing nozzles [48]. Another critical factor that may cause defects in the nanofiber structure is the density of the surface charge. Any change in the surface charge density may also affect the nanofiber morphology. Theron et al. revealed that the electric current and flow rate are related. They studied the effects of the flow rate and surface charge density using various polymers, including polyacrylic acid (PAA), PEO, polyvinyl alcohol (PVA), polycaprolactone (PCL), and polyurethane (PU). In the PEO polymer, an increase in the flow rate simultaneously increased the electric current and decreased the surface charge density. Reduction of the surface charge density allowed the merging of electrospun nanofibers during their flight toward the collector [49,50].

### 3.2. Effect of Distance between the Needle and Collector

The distance between the collector and metallic needle tip and plays an essential role in determining the electrospun nanofiber morphology. Similar to the applied electric field, the flow rate, and the viscosity, the distance between the collector and metallic needle tip also varies with the polymer system. The morphology of the nanofiber is influenced by the distance because it depends on the deposition, evaporation rate, time, and instability interval or whipping [51]. Consequently, a critical distance needs to be maintained to formulate smooth and uniform electrospun nanofibers. Any changes on either side of the critical distance will affect the morphology of the nanofibers [52]. Several researchers have studied the effect of the distance between the collector and needle tip, then concluded that defective and large-diameter nanofibers are formed when this distance is kept small, whereas the diameter of the nanofiber is decreased as the distance was increased [51]. There are also cases where no effect on the morphology of the nanofiber was observed with a change in the distance between the collector and metallic needle [53].

## 4. Classification of Nanoparticles and Silver Nanoparticles in Wound Healing

Many nanomaterials are loaded with therapeutic agents that stimulate the wound healing process, including polymeric nanoparticles, inorganic nanoparticles, and lipid nanoparticles. Polymeric nanoparticles are colloidal biocompatible systems that attract significant attention in biomedicine and bioengineering [54]. When drugs are incorporated into these nanoparticles, they are protected from biodegradation by the wound proteases and are released in a sustained and controlled manner to reduce frequent administration. Presently, most polymeric nanoparticles are formulated from PLGA, chitosan, gelatin, alginate, etc. Nanoparticles that are prepared from these polymers can be further loaded in bioactive wound dressings [55]

Lipid nanoparticles are mostly designed with lipid molecules or physiological lipids, and their formulation does not require the use of toxic organic solvents [55]. Lipid nanoparticles are divided into two groups: nanostructured lipid nanocarriers (NLCs) and solid lipid nanoparticles (SLNs). The potential of nanoparticles for topical cosmetic or therapeutic purposes has been moderately exploited [55]. NLCs are prepared using oil, while SLNs are prepared using organic solvents. SLNs are hydrophobic, and the drug is loaded in its core resulting in a slow drug release. They are also characterized by large surface area and low toxicity [46].

Inorganic nanoparticles are composed of inorganic materials, such as ceramic nanoparticles, metallic nanoparticles (metal oxide nanoparticles), carbon-based nanoparticles, etc. [56]. These nanoparticles demonstrate attractive advantages in wound healing management and superior bactericidal effect. The activity and toxicity of inorganic nanoparticles depend on key features, such as dimension and architecture (smaller particles are more biologically active), surface charge, poly-dispersity index, and surface functionalization. Therefore, the combination of inorganic nanoparticles is essential for synergistic stimulating therapeutic effect. Examples of metallic nanoparticles include iron nanoparticles, copper nanoparticles, zinc nanoparticles, silver nanoparticles, etc. The silver nanoparticles are widely employed to improve the antimicrobial properties of bioactive wound dressings [14].

There are several advantages of Ag nanoparticles in wound healing application which include low systemic toxicity and antibactericidal effect with the capability to prevent the development of drug resistance [24,57,58]. However, despite the good therapeutic outcomes of silver compounds, silver sulfadiazine and silver nitrate cause argyria when used in topical formulations applied over an extended duration. Furthermore, formulations incorporated with silver can cause toxicity to the epithelial and fibroblast cells. Despite the above-mentioned limitations, silver sulfadiazine is the most commonly used topical antibacterial agent [59]. The bactericidal effects of Ag nanoparticles have been known since ancient times, and they are effective against a wide broad spectrum of Gram-positive, Gram-negative, and even antibiotic-resistant bacteria [60]. The antimicrobial effect of Ag nanoparticles is due to disruption of bacterial cell membrane proteins and interaction with DNA [60].

## 5. Nanofibers Loaded with Silver Nanoparticles

Nanofibers are regarded as ideal wound dressing materials with diameters in the nanometers range, and they are suitable for the treatment of chronic wounds because of their drug-delivery capacity [61]. The nanofibers that are formulated by electrospinning method, display interesting properties such as high porosity rates, gas permeation, and possess a high surface-area-to-volume ratio [62,63,64]. These properties result in enhanced skin regeneration, cell respiration, moisture regeneration, haemostasis, and absorption of wound exudates [65]. Nanofibers mimic the extracellular matrix (ECM), thereby improving the proliferation of epithelial cells and the development of new tissues at the wound site [66,67,68]. Their nanometer diameter of nanofibers promotes haemostasis of damaged tissues, cell respiration, promote dermal drug delivery, and enhance fluid absorption, and high-gas permeation, thereby preventing microbial infections [69].

Ganesh et al. formulated PVA-chitosan composite electrospun nanofibers co-encapsulated with Ag nanoparticles and sulfanilamide for synergistic wound-healing effect. The successful formulation of Ag nanoparticle-loaded nanofibers physicochemical properties was confirmed by Fourier transform infrared (FTIR) spectroscopy and X-ray diffraction (XRD) analysis. The scanning electron microscopy (SEM) images of the nanofibers revealed continuous and smooth morphology with an average fiber diameter of 150 nm, which is suitable for the encapsulation of Ag nanoparticles for the treatment of microbial infected wound healing. The swelling analysis displayed that the degree of swelling in PVA–chitosan nanofibers was influenced by Ag nanoparticles/sulphanilamide quantity. The feature mentioned above reveals that Ag nanoparticles/sulphanilamide was crosslinked with the polymer matrix via hydrogen bonding to hinder water uptake and the swelling of the drug-encapsulated nanofibers [70].

The in vitro drug release showed that the drug release was rapid from the nanofibers in the first initial hours, followed by relatively slow release. The antibacterial studies demonstrated a significantly increased zone of inhibition of PVA–chitosan nanofibers loaded with Ag nanoparticles and sulfanilamide against *Staphylococcus aureus*, *Escherichia coli,* and *Pseudomonas aeruginosa* compared to the plain nanofiber, suggesting that the combination of Ag nanoparticles and sulphanilamide incorporated in the polymer nanofibers enhanced the antimicrobial effect of the scaffolds significantly. The in vivo wound healing evaluation using rat model revealed wound closure of the PVA–chitosan nanofibers gradually increased similarly as the co-loaded nanofibers and reached 90.76 ± 4.3% after 7 days. In contrast, the wound closure of the control was 55.26 ± 3.5% after 20 days [70].

Kharaghani et al. formulated electrospun PVA-chitosan nanofibers loaded with Ag and copper nanoparticles. Their physicochemical properties were confirmed by X-ray diffraction (XRD) analysis and attenuated total reflectance spectroscopy (ATR). The in vitro antimicrobial analysis showed that the nanofibers containing Ag nanoparticles displayed improved antibacterial properties compared to the copper nanoparticles [71]. Alipour et al. fabricated nanofibers that are based on PVA-pectin loaded with Ag nanoparticles for wound healing applications. The successful formulation of PVA-pectin nanofibers was confirmed by energy dispersive X-ray analysis (EDS), XRD analysis, and FTIR spectra analysis. The mechanical property analysis of nanofibers exhibited elongation at break, Young’s modulus, and tensile strength of 260.5 ± 8.2%, 7.7 ± 0.21 Mpa, and 63.4 ± 3.3 Mpa, respectively. The in vitro cytotoxicity evaluation of the polymeric nanofibers employing MTT assay showed significantly high cell viability of HSF-PI 18 fibroblast cells confirming non-toxicity of the nanofibers with high antibacterial effects against *Escherichia coli* and *Pseudomonas aeruginosa*, and *Staphylococcus aureus* strains in vitro. The in vivo wound healing experiments using wounds on the back of white rabbits demonstrated that the wound healing process of the wounds treated with nanofibers loaded with Ag nanoparticles was significantly accelerated compared to those treated with the plain nanofibers. The rate of wound closure was significant for the polymeric nanofibers encapsulated with a higher amount of Ag nanoparticles [72].

Aadil et al. formulated PVA-lignin electrospun nanofibers loaded with Ag nanoparticles as potential antibacterial wound dressings. The thermal gravimetric analysis (TGA) of nanofibers exhibited a maximum weight loss percentage between 200 and 300 °C, which is due to the decomposition of C-C bonds in lignin. The in vitro antimicrobial analysis of PVA-lignin electrospun nanofibers loaded Ag nanoparticles showed an inhibition zone of 1.1 ± 0.05 and 1.3 ± 0.08 cm against *E. coli* and *B. circulans*, respectively, revealing that Ag nanoparticles encapsulated polymeric nanofibers had good antibacterial efficacy, which is due to its spherical shape, small size and large surface area [73].

Balakrishnan and Thambusamy prepared PVA-β-cyclodextrin nanofibers loaded with Ag nanoparticles and riboflavin for wound healing applications [74]. The in vitro cytotoxicity evaluation employing MTT assay in HEK-293 cells demonstrated cell viability of more than 90% in the presence of dual drug-loaded PVA-β-cyclodextrin nanofibers indicating their excellent biocompatibility that resulted in no sign of erythema and edema on the rat skin in vivo. The in vivo wound healing assessments using wounds in Wister rats showed that the percentage of wound closure was 63%, 75%, 87% and 98% for the plain PVA-β-cyclodextrin nanofibers, PVA-β-cyclodextrin nanofibers loaded with Ag nanoparticles, PVA-β-cyclodextrin nanofibers loaded with riboflavin, and PVA-β-cyclodextrin nanofibers loaded with Ag nanoparticles and riboflavin, respectively [74].

Yang et al. designed Janus nanofiber wound dressings from PVP and ethyl cellulose via a side-by-side electrospinning technique. The nanofibers were loaded with Ag nanoparticles and ciprofloxacin. The successful preparation of the dual drug-loaded Janus nanofibers was confirmed by FTIR and XRD. The in vitro drug release revealed a controlled release pattern of the drug from the nanofibers. The in vitro antibacterial analysis demonstrated the antibacterial activity of Janus nanofibers against both *S. aureus* and *E. coli,* indicating a synergistic action of both the Ag nanoparticles and ciprofloxacin [75]. Lee et al. formulated electrospun chitosan-based nanofibers encapsulated with Ag nanoparticles for bacteria-infected wound care. The SEM images displayed homogeneous nanofibrous structures with uniform morphology and mean fiber diameters of 460 ± 80 nm, 126 ± 28 nm for plain nanofibers, and nanoparticle-loaded nanofibers, respectively. The in vitro antimicrobial evaluation of nanofibers demonstrated a high zone of inhibition against *P. aeruginosa* and *Methicillin-resistant Staphylococcus aureus* (MRSA), suggesting that the chitosan-based nanofibers encapsulated with Ag nanoparticles are potential therapeutics for the treatment of infected wounds [76].

Khan et al. synthesized nanofibers that are based on cellulose acetate loaded with Ag-sulfadiazine. TGA analysis demonstrated the thermal degradation of nanofibers loaded with Ag-sulfadiazine that increased with the addition of Ag-sulfadiazine in the nanofibers. The FTIR results showed strong chemical interactions between Ag-sulfadiazine and cellulose acetate in the nanofibers. The water contact angle studies displayed that these nanofibers had hydrophilic nature with water contact angles of 28° and 50° that is suitable for wound dressing material, but the water-absorbing property of nanofibers was reduced as the amount of Ag-sulfadiazine increased. The antibacterial analysis using the agar disk diffusion method showed that these nanofibers possess superior antibacterial activity against *E. coli* and *B. subtilis* [77]. El-Aassar et al. formulated polygalacturonic-hyaluronic acid nanofibers incorporated with Ag nanoparticles for wound healing applications. The mechanical property analysis demonstrated that the tensile stress of Ag nanoparticles-loaded nanofibers (∼4.1 MPa) was almost two times stronger than the plain (∼1.9 MPa). The nanofibers loaded with Ag nanoparticles were very effective against *B. subtilis*, *S. aureus*, and *E. coli,* while the pure nanofibers did not show any significant antibacterial effects. The in vivo wound healing experiments revealed the wound closure capability of Ag nanoparticle-nanofibers and plain nanofibers on the treated wounds was significant from day 8th day. Re-epithelisation was substantial on day 14, whereas the Garamycin^®^ cream and control (saline) wound closure took more than 14 days [78].

Shi et al. prepared PU-g-PEG nanofibers loaded with Ag nanoparticles. The haemolysis experiments showed that the PU and Ag nanoparticle-loaded PU nanofibers displayed a higher haemolysis ratio compared to the nanofibers grafted with PEG. The cell adhesion experiments showed that the number of the attached red blood cells (RBCs) were higher on the nanofibers, although the portion of these RBCs underwent complete deformation indicating the toxicity of Ag nanoparticles to the RBCs. The in vitro antimicrobial analysis of nanofibers demonstrated significant activity against *S. aureus* and *E. coli* strains of bacteria [79]. Dubey et al. formulated PEO–PCL blended nanofibers loaded with Ag nanoparticles. The TGA results displayed no weight loss up to 160 °C. The weight loss mostly happened in the temperature between 160 and 360 °C, with an insignificant change at temperatures higher than 360 °C. The in vitro drug release profiles of nanofibers under physiological conditions displayed initial burst release of Ag nanoparticles for the first 24 h followed by a constant slow and sustained release. A high inhibition zone was visible after 4 h, indicating a strong antibacterial effect of the membranes against antibiotic-resistant *E. coli* [80].

## 6. Nanofibrous Materials for Wound Management

### 6.1. Nanofibrous Mats Loaded with Silver Nanoparticles

Nanofibrous mats are wound dressing materials that are mostly prepared from biopolymers and synthetic polymers, including cellulose, collagen, gelatin, chitosan, chitin, alginate, PU, PEG, PCL, and loaded with bioactive agents such as silver, ciprofloxacin, and gentamycin [81]. The wound healing response is stimulated by the nature of the biomaterials employed for the formulation. Electrospun nanofibrous mats are gaining interest currently in wound healing research as it allows easy gas exchange at the wound environment. They are also useful for the incorporation of hydrophobic bioactive agents and display sustained and controlled drug release of the loaded bioactive molecule [40]. These wound dressings also mimic the skin’s ECM, and hence it can accelerate the wound healing process and prevent bacterial infections (Table 2) [41,82].

Aktürk et al. prepared antibacterial PVA nanofibrous mats loaded with starch and coated with Ag nanoparticles. The SEM micrographs of PVA nanofibrous mats displayed wrinkled and porous morphology, which is important for the sustained release pattern of Ag nanoparticles. The average fiber diameters of the plain PVA nanofibrous mats and nanoparticle-loaded PVA were found to be 163 ± 42 nm and 141 ± 37 nm, respectively. The in vitro antimicrobial analysis showed potent growth inhibitory and superior antibacterial effects against *E. coli* and *S. aureus* when incubated with PVA nanofibrous mats loaded with starch and coated with Ag nanoparticles [83]. Dashdorj et al. designed zein electrospun nanofibrous mats incorporated with Ag nanoparticles. The mechanical analysis of the nanofibrous mats showed a high tensile strength of 2 Mpa. The cytotoxicity analysis using CCK assay showed an increased cell proliferation of fibroblast cells on the 3rd day and 6th day for the plain and nanoparticle-loaded mats indicating that the encapsulation of Ag nanoparticles did not affect the excellent biocompatibility of zein nanofibrous mats. The cell adhesion analysis demonstrated that the fibroblast cells were well attached to the electrospun zein nanofibrous mats indicating a good interaction between the zein nanofibrous mats and the cells. This also shows that the fibroblast cells’ capability to grow in the nanofibrous mats, and capability to accelerate the wound healing process. The antimicrobial analysis showed a superior zone of inhibition around and within the nanofibrous mats loaded with ZnO nanoparticles against *E. coli* and *S. aureus* after 24 h of incubation, indicating their capability to treat bacteria-infected wounds [84].

Kim et al. synthesized polyurethane (PU) electrospun nanofibrous mats encapsulated with Ag nanoparticles for wound healing applications. The SEM images of the nanofibrous mats encapsulated with Ag nanoparticles exhibited smooth nanofiber morphology and randomly oriented nanofibers with uniform average diameters of 427.38 ± 204.47 nm. The cell adhesion analysis of the mats displayed high fibroblast cell attachment, and the water angle contact evaluation showed that the encapsulation of nanoparticles improved the hydrophilicity nature. The in vitro antimicrobial test on the nanofibrous mats showed high growth inhibition of *E. coli* and *S. aureus* revealing good antibacterial efficacy [85]. Esmaeili et al. formulated PU-cellulose acetate electrospun nanofibrous mats co-encapsulated with Ag nanoparticles/reduced graphene oxide and curcumin. The water vapour transmission studies demonstrated a significant WTVR of 1343.4 ± 31.5 g/m^2^/day. The in vivo wound healing experiments of co-loaded nanofibrous mats on a male rat model displayed an accelerated healing rate than the single drug-loaded mats. The co-loaded nanofibrous mat samples revealed a complete wound closure of 100% on the 15th day, 93% for single drug-loaded nanoparticles, and 78% for the control [86].

Kohsari et al. fabricated PEO-chitosan antibacterial nanofibrous mats incorporated with Ag nanoparticles. The mechanical experiments of PEO-chitosan nanofibrous mats showed very high flexibility with no fracturing upon 180° bending. The in vitro drug release studies showed the release of Ag nanoparticles was a burst release within the initial 8 h followed by a slow and sustained release. The in vitro antimicrobial analysis of Ag nanoparticle-loaded nanofibrous mats demonstrated more than 99% antibacterial activity against *E. coli* and *S. aureus* [87]. Abdelgawad et al. developed PVA-chitosan nanofiber mats incorporated with Ag nanoparticles. The in vitro antibacterial analysis demonstrated superior bactericidal efficacy of the Ag nanoparticles in the composite PVA-chitosan nanofibrous mats against *E. coli*, indicating that these mats are suitable for the treatment of bacteria-infected wounds.

Mohan et al. prepared electrospun double-layer nanofibrous mats composed of an upper layer of PVA and chitosan loaded with Ag nanoparticles. The in vitro antimicrobial evaluation of the prepared PVA-chitosan nanofibrous mats against *S. aureus*, *E. coli*, *P. aeruginosa*, and *C. albicans* using the disk diffusion method demonstrated superior broad-spectrum antibacterial activity, suggesting further attention for wound-healing applications. *Srivastava* et al. synthesized silk fibroin nanofibrous mats incorporated with Ag nanoparticles. The porosity analysis of Ag nanoparticles loaded nanofibrous mats displayed an average porosity of 80%, which can play a very significant role in the proliferation, adhesion, and growth of cells. The fluid uptake studies showed the water uptake of Ag nanoparticle-loaded nanofibrous mats was 70% with a WVTR of 2300 g/m^2^/day. The in vitro biodegradation experiments of the nanofibrous mats loaded with Ag nanoparticles after incubation in PBS with and without trypsin enzyme exhibited a controlled rate of degradation after 30 days of incubation. The SEM micrographs clearly showed that the nanofibrous mat network was destroyed, and only a few nanofibers remained after 30 days, indicating that the nanofibrous materials can be very useful in skin regeneration for injured skin [88].

Du et al. synthesized PVA nanofibrous mats embedded with ascorbyl palmitate and loaded PCL/silver nanoparticle as wound dressings. The in vitro cytotoxicity analysis using NIH-3T3 fibroblast cells showed that the encapsulation of ascorbyl palmitate could significantly reduce the toxic effect of Ag nanoparticles on cell proliferation. The in vitro antimicrobial analysis of the nanofibrous mats displayed a high antimicrobial effect against *S. aureus* and *E. coli*. The in vivo wound healing studies employing the male mice model showed that PVA nanofibrous mats embedded with ascorbyl palmitate loaded PCL/silver nanoparticle revealed the highest wound-closure ratio of 99% on day 18. The wound-contraction ratio of the PCL/silver nanoparticle-loaded mats, PCL/ascorbyl palmitate-loaded mats, and plain nanofibrous mats were 94%, 91% and 85%, respectively [89]. Wang et al. formulated electrospun keratin/PU nanofibrous mats loaded with Ag nanoparticles. The in vitro antibacterial evaluation showed that keratin/PU nanofibrous mats loaded with Ag nanoparticles had better antibacterial activity against *E. coli* and *S. aureus*. The in vivo wound closure studied using female Sprague–Dawley rats showed that the nanofibrous mats remarkably accelerated the wound healing process compared to the gauze sponge wound dressing (control) [90].

Maharjan et al. developed PU-zein hybrid nanofibrous mats loaded with Ag nanoparticles. The successful formulation of the hybrid nanofibrous mats loaded nanoparticles was confirmed by FTIR, XRD, and TGA analysis. The in vitro cell proliferation experiments using CCK assay showed that the developed hybrid nanofibrous mats could enhance the cell attachment and cell proliferation when incubated with NIH-3T3 fibroblasts at physiological conditions compared to the plain PU nanofibrous mats. The in vitro antimicrobial studies showed that the plain PU mats and PU-zein hybrid nanofibrous mats did not reveal the antibacterial efficacy. On the other hand, a great inhibition zone was detected for fabricated PU-zein hybrid nanofibrous mats loaded with Ag nanoparticles [91]. Ballesteros et al. PCL-based electrospun nanofibrous mats incorporated with Ag nanoparticles-nanogels. The in vitro drug release profile showed the capability of PCL nanofibrous mats to release Ag nanoparticles in a controlled pattern after the laser irradiation was applied. The in vitro antibacterial experiments of nanofibrous mats incorporated with Ag nanoparticles-nanogels demonstrated superior bactericidal performance with inhibition zone diameter of 1.8 ± 0.5 mm and 2.6 ± 0.3 mm for *E. coli* and *S. aureus*, respectively, indicating the nanofibrous mats is a potential wound dressing for the management of bacteria-infected wounds [92].

Wadke et al. reported PVA-starch-based nanofibrous mats encapsulated with Ag nanoparticles. The SEM images of the nanofibrous mats were porous with a dense meshwork of submicron fibers, non-woven, randomly arranged in a planar orientation. The interconnected pores were small and ranged between 50–60 nm, and bound by different fibers, which is very useful to promote cell migration and proliferation. Water retention analysis displayed that the water retention feature of the PVA-starch-based nanofibrous mats increased till the 5th day followed by a sudden decrease. The in vitro antibacterial efficacy of the nanofibrous mats loaded with Ag nanoparticles was higher in *E. coli* than *S. aureus*. The biodegradation experiments in vitro demonstrated that PVA-starch-based nanofibrous mats incubated in PBS solution containing lysozyme showed the highest weight loss of approximately 19.24 ± 0.57% in the first week compared with plain PBS at pH 7.4, indicating that the mats are biodegradable and can significantly accelerate skin regeneration [93].

Lin et al. designed electrospun PVA-based nanofibrous mats impregnated with Ag nanoparticles [94]. The in vitro antibacterial experiments exhibited outstanding growth inhibition efficacy against *S. aureus* and *E. coli.* Ag nanoparticle-loaded nanofibrous PVA mats prepared by Destaye et al. also demonstrated excellent antimicrobial efficacy against *E. coli*, suggesting that these nanofibrous scaffolds are potential antibacterial wound dressings [95]. Li et al. prepared PVA-chitosan nanofibrous mats incorporated with Ag nanoparticles for wound healing applications. The SEM micrographs demonstrated the smooth morphology of the nanofibers with a mean fiber diameter that ranged between 130–192 nm. The nanofibrous membranes loaded with Ag nanoparticles showed significantly high growth inhibition of *S. aureus* and *E. coli* bacteria. The in vivo wound healing studies of the electrospun mats loaded with Ag nanoparticles employing full-thickness circular wounds in Sprague Dawley rats exhibited an accelerated wound closure rate than the gauze used as the control [96].

Eghbalifam et al. formulated gum Arabic nanofibrous mats encapsulated with Ag nanoparticles. The SEM micrographs showed that the mats possessed randomly oriented fibers morphology with a porous structure, increasing the surface-to-volume ratio. The in vitro antibacterial experiments showed a higher zone of inhibition for Ag nanoparticle-loaded nanofibrous mats than the plain nanofibrous mats against *S. aureus*, *E. coli P. aeruginosa* [97]. Zhang et al. designed PLA-based electrospun nanofiber mats loaded with silver nanoparticles for wound healing applications. The in vitro antimicrobial evaluation showed a 99.9% reduction for the nanoparticles-loaded mats against *E. coli* and *S. aureus*, which was enhanced compared to the blank electrospun mats. The in vivo wound closure analysis revealed that the wound closure was accelerated with a healing ratio of 99.9% for Ag nanoparticles-loaded mats, higher than the pure nanofibrous mats (80.1%) [98].

### 6.2. Nanofibrous Membranes Loaded with Silver Nanoparticles

Nanofibrous membranes are a versatile class of nanomaterials that have been recognized as promising materials because of their excellent properties such as small diameters, high porosity, high surface-to-volume ratio, and outstanding pore interconnectivity [99,100]. Extensive research studies have been conducted in previous years to assess electrospun nanofibrous membranes in terms of membrane technology, which result from the advantages such as the cost and productivity compared to the complicated bottom-up approach, flexibility of the membranes, and the potential of scaling up for industrial production (Table 2) [101,102]. Shao et al. prepared chitosan nanofibrous membranes incorporated with Ag nanoparticles using the electrospinning method. The SEM micrographs demonstrated a uniform fibrous structure for all prepared membranes with a mean fiber diameter of ~200 nm. At the same time, the TEM images showed electron-dense Ag nanoparticles within the fibers. The mechanical analysis of Ag nanoparticles loaded nanofibrous membranes showed significantly higher tensile strength, Young’s modulus, and elongation at break of 22.21 ± 5.03 MPa, 841 ± 165 MPa, and 6.32 ± 1.84%, respectively [103].

The in vitro drug release studies at 37 °C phosphate buffer saline (PBS) demonstrated a slow and sustained silver release pattern, and the quantity of silver released was not influenced by the amount of Ag nanoparticles encapsulated in the nanofibrous membranes. The in vitro antibacterial analysis after immersion of the membranes with *S. aureus* was up to 28 days and showed a high zone of inhibition (ZOI) and the diameter of ZOI decreased with an increase in the incubation period. The in vivo wound healing studies using rat full-thickness skin showed that the Ag nanoparticles impregnated chitosan nanofibrous membranes accelerated a healing process with complete wound closure within 2 weeks compared to the sham control [103]. Cheng et al. prepared PVA-co-PE nanofibrous membranes impregnated with Ag nanoparticles. The antibacterial assessments of the nanoparticles loaded membranes showed antibacterial efficiency of 99.99% against *E. coli* and *S. aureus* within 60 min [104].

Thomas et al. reported PCL-based electrospun nanofibrous membranes incorporated with Ag nanoparticles for bacteria-infected wounds [105]. The successful preparation of the nanoparticle-loaded nanofibrous membranes and the physicochemical properties were confirmed by Fourier transform infrared (FTIR) spectroscopy and X-ray diffraction (XRD) analysis. The SEM results of Ag nanoparticle-loaded membranes showed smooth morphology, suggesting that the nanoparticles were homogeneously dispersed in the nanofibrous membranes. The encapsulation of Ag nanoparticles in the PCL membranes did not influence the morphology of the membranes. The water contact angle analysis of the membranes using the sessile drop technique showed that the encapsulation of Ag nanoparticles changed the hydrophobicity nature of the plain PCL membranes to hydrophilicity nature by showing a water contact angle of 73°. The antimicrobial analysis showed that the formulated nanofibrous membranes loaded with Ag nanoparticles possessed significant antibacterial efficacy against *S. epidermidis* and *S. haemolyticus* compared to the neat nanofibrous membranes (control). All the above results revealed that Ag nanoparticles could be potential wound dressings [105]. Song et al. developed antibacterial PEO-chitosan nanofibrous membranes loaded with Ag nanoparticles and chlorhexidine. The in vitro drug release profile showed Ag^+^ was released in a sustained manner for approximately a month, regardless of the presence of chlorhexidine. The in vitro cytotoxicity studies revealed high cell viability of human foreskin fibroblasts on the membranes loaded with either chlorhexidine and silver nanoparticles, suggesting that these nanofibrous membranes are non-toxic that can be used as biocompatible wound dressings [106].

Shi et al. designed antibacterial PEO-carboxymethyl cellulose electrospun nanofibrous membranes functionalized with Ag nanoparticles [107]. The SEM images of the Ag nanoparticle electrospun membranes were a smooth morphology with an average fiber diameter of 89 ± 23 nm. The TGA analysis of the nanofiber membranes demonstrated that the electrospun membranes were thermally degraded at 316 °C, due to the decomposition of carboxymethyl cellulose. In contrast, the second weight loss at 417 °C was associated with the thermal decomposition of PEO. The swelling analysis of nanofibrous membranes displayed a swelling ratio of 700% after 12 h, while after 24 h, it increased to 1400%. This high swelling ratio was due to the physical and chemical structure of the PEO-carboxymethyl cellulose electrospun nanofibrous membranes functionalized Ag nanoparticles. The in vitro antimicrobial experiments demonstrated 100% antibacterial efficacy of the Ag nanoparticle-loaded nanofiber membranes against *S. aureus* and *E. coli*, indicating their significant potential application in bacteria-infected wounds [107].

Amina et al. designed olive oil/PU electrospun nanofibrous membranes decorated with Ag nanoparticles skin injuries. The successful formulation of the nanofibrous loaded with nanoparticles was confirmed by energy-dispersive X-ray (EDX) and X-ray diffraction (XRD) characterization. The in vitro cytotoxicity analysis using MMT assay showed that the nanofibrous membranes are non-toxic when incubated with fibroblast cells. The antibacterial analysis on the olive oil/Ag-PU electrospun nanofibrous membranes loaded with Ag nanoparticles showed high growth inhibition of *E. coli* and *S. aureus* that are common in burn wound, indicating that the nanofibrous scaffolds are endowed with good bactericidal properties due to the introduction of olive oil and Ag nanoparticles. The nanofibrous scaffolds’ capability to reduce wound contamination and accelerate the wound healing process was significant [108]. Shalumon et al. prepared PEG-PCL nanofibrous membranes encapsulated with Ag nanoparticles. The in vitro cytotoxicity evaluation of the PEG-PCL nanofibrous membranes encapsulated with Ag nanoparticles displayed high cell viability indicating good biocompatibility of the nanofibrous scaffolds with excellent antibacterial effect against *E. coli* and *S. aureus* [109]. Alippilakkotte et al. prepared and evaluated PLA-based nanofibrous membranes loaded with Ag nanoparticles. The mechanical analysis of the nanofibrous membranes showed tensile strength that enhanced from 0.32 ± 0.04 to 0.82 ± 0.05 MPa when the Ag nanoparticle concentration was increased from 0 to 2 wt%. The water vapor transmission analysis showed that these membranes had WVTR of 2237.53 ± 165 g/m 2.24 h, indicating that these nanofibers can maintain optimal moisture for the proliferation and functions of the fibroblasts and epidermal cells. The in vitro cytotoxicity experiments of nanofibrous membranes demonstrated more than 93.96% cell viability of L-929 mouse fibroblast cells when immersed with nanofibers displaying excellent biocompatibility as ideal wound dressing material with good antibacterial activity against *E. coli* and *S. aureus* [110].

**Table 2:** Some nanaofibrous materials reported as potential wound dressings.

Nanaofibrous Materials	Polymers	Bioactive Agents Loaded with Ag Nanoparticles	Outcome	The Concentration of Ag Nanoparticles	References
Nanofibrous mats	Polyvinyl Alcohol (PVA)	Starch	-Sustained release of Ag nanoparticles. -Wrinkled and porous morphology. -Antibacterial effect against *E. coli* and *S. aureus*.	1 wt%	[74]
	Chitosan	-	-Excellent antimicrobial activity against *P. aeruginosa* and *Methicillin-resistant* *Staphylococcus aureus* (*MRSA*).	0, 0.7, 1.3, 2, 4 wt%	[76]
	Cellulose acetate	-	-Good biocompatibility. Good antimicrobial activity against *E. coli* and *B. subtilis.*	0.125, 0.25, 0.37, 0.5 wt%	[77]
	-Polyethylene oxide -polycaprolactone	-	Good antimicrobial activity.	1–3 wt%	[80]
	Polycaprolactone and poly vinyl alcohol	ascorbyl palmitate	-Antimicrobial activity of Ag nanoparticle-loaded mats revealed a 99% reduction against *E. coli* and *S. aureus.*	2 wt%	[89]
Nanofibrous membranes	Polyvinyl alcohol	-	-Good biocompatibility.	1% *w/v*	[94]
	Polyvinyl alcohol	-	-Potent antibacterial efficacy against *E. coli*.	-	[95]
	Poly vinyl alcohol, chitosan	-	-Antibacterial efficacy against *S. aureus* and *E. coli*. Promotes the formation of granulation tissue.	1,2, 3, 4, 5 wt%	[96]
	Gum Arabic, poly caprolactone, poly vinyl alcohol	-	-Good antibacterial activity against *S. aureus*, *E. coli*, *P. aeruginosa* and *C. albicans*.	3 wt%	[97]
	Poly lactic acid	-	Good antimicrobial efficacy against *E. coli P. aeruginosa*, *S. aureus*, *M.* smegmatis in vitro with bacteria inhibition rate of more than 95.0%. Accelerated wound healing of 99.9% of *S. aureus*-infected wound.	-	[98]
	Poly (lactide-co-glycolide)	-	-supports cell attachment, proliferation, and viability of human osteoblast-like MG-63 cells, in vitro.	-	[99]
	Chitosan	-	-Sustained drug release. -Accelerated wound healing in vivo.	12, 60 mg	[103]
	Polyvinyl alcohol, polyethylene	-	Potent antibacterial activity of 99.9%.	10–100 mL	[104]

## 7. Conclusions

The incorporation of nanoparticles in the electrospun nanofibers or nanofibrous scaffolds enhanced the hydrophilic nature. The in vitro and in vivo experimental studies on electrospun nanofibers scaffolds loaded with Ag nanoparticles revealed their excellent antibacterial properties. The electrospun nanofibers scaffolds loaded with Ag nanoparticles also exhibited good biocompatibility, high porosity, non-toxicity, biodegradation, and excellent antibacterial efficacy. These nanofibers scaffolds have gained popularity globally due to their hydrophilic properties and sustained release pattern. They play a significant role in wound dressing by enhancing and accelerating the wound healing process. Furthermore, the combination of silver nanoparticles with other antibacterial agents revealed synergistic antibacterial effects. The reports from different researchers have demonstrated the efficacy of silver nanoparticles in wound healing and skin regeneration. There is no doubt that more research will results in scaffolds with distinct features suitable for the management of chronic wounds. However, the synthesis of silver nanoparticles via green chemistry is a good approach because it does not require the use of toxic reagents. Loading silver nanoparticles prepared by green chemistry into wound dressings is a potential approach to develop wound dressings that are non-toxic and biocompatible.

## Data Availability

Not applicable.
